# Male sterility significantly elevates shape variation and fluctuating asymmetry of zygomorphic corolla in gynodioecious *Glechoma hederacea* (Lamiaceae)

**DOI:** 10.1093/aobpla/plab013

**Published:** 2021-04-08

**Authors:** Jiri Neustupa, Katerina Woodard

**Affiliations:** Department of Botany, Faculty of Science, Charles University, Prague 12843, Czech Republic

**Keywords:** Bilateral symmetry, floral biology, geometric morphometrics, *Glechoma hederacea*, gynodioecy

## Abstract

Female flowers of gynodioecious plants usually have smaller corollas than bisexual flowers. This difference is explained as a developmental consequence of stamen abortion and as a result of stronger selection for larger corolla size in hermaphroditic flowers that solely ensure male function within populations. This study evaluated whether the size difference of zygomorphic corollas in a widely distributed gynodioecious herb *Glechoma hederacea* is accompanied by variation in shape and bilateral fluctuating asymmetry of sexually differentiated flowers. Geometric morphometric analyses of bilateral symmetry in the shapes of corolla lower lips showed that male-sterile flowers were significantly more plastic and asymmetric, implying that they may be subjected to weaker stabilizing selection for corolla shape in comparison to hermaphrodites. These results illustrated that sexual differentiation may be an important factor contributing to bilateral fluctuating asymmetry in the shape of zygomorphic flowers.

## Teaser

Gynodioecy is dimorphic sexual system where female and hermaphrodite flowers are borne on different individuals. We used geometric morphometrics of bilateral symmetry in corolla shapes of gynodioecious *Glechoma hederacea* (Lamiaceae) to test for the differences in shape plasticity and fluctuating asymmetry between the sexually differentiated flowers. The analyses showed that male-sterile flowers were significantly more plastic and asymmetric, implying that they may be subjected to weaker stabilizing selection for corolla shape in comparison to hermaphrodites. These results illustrated that sexual differentiation may be an important factor contributing to bilateral fluctuating asymmetry in the shape of zygomorphic flowers.

## Introduction

The sexually differentiated flowers of angiosperms typically diverge in size (Barrett and Hough 2013). Darwin (1877) was one of the first scientists to notice that the corolla of female flowers is usually reduced in size compared to hermaphroditic or male flowers. It was proposed that this dimorphism is largely a consequence of stamen abortion in purely pistillate flowers, leading to corolla reduction due to the close developmental relationship between the stamens and corolla (Plack 1957; Delph 1996; Theißen and Rümpler 2018). However, Baker (1948) showed that dimorphism in corolla size usually does not occur in wind-pollinated species but is much more typical for sexually differentiated taxa pollinated by animals. In addition, it was repeatedly illustrated that polleniferous flowers, typical of a larger corolla, attracted more pollinators than their male-sterile counterparts within the same species (Bell 1985; Delph and Ashman 2006). Therefore, it is possible that the ultimate evolutionary cause of corolla size dimorphism may be stronger selective pressure for the visual attraction of pollinators to polleniferous flowers, which elevates male fitness by increasing the pollen load transported among flowers (Delph 1996). Conversely, the fitness of female plants is only affected by the receipt of fertile pollen grains, which typically requires a comparatively smaller number of pollinator visits.

Gynodioecy is one of the patterns of sexual differentiation describing the coexistence of individuals with hermaphrodite and female flowers (Dufay *et al.* 2014; Rivkin *et al.* 2016). It has evolved multiple times across the angiosperm phylogenetic tree. From an evolutionary point of view, gynodioecy is one of the pathways between ancestral hermaphroditism and fully developed dioecy (Barrett 2002; Dufay *et al.* 2014). In gynodioecious plants, the male function is solely assured by individuals with hermaphroditic flowers. The purely pistillate flowers of female plants often contain aborted sterile staminodes (Darwin 1877). The differences in corolla size between the sexes in gynodioecious species are well-documented (Darwin 1877; Delph 1996; Kamath *et al.* 2017). However, there is much less information on possible differences in corolla shape between hermaphroditic and female flowers. Different selective pressures on these sexually separated individuals, as well as the differences in their ontogeny due to the abortion of stamens in female plants, might lead to systematic variation in corolla shape between the sexes. In addition, corollas of hermaphroditic and male-sterile flowers could also differ in their developmental instability (DI), which is defined as the shape variation among morphological structures with the same expected target phenotype resulting from imprecision of developmental processes (Graham *et al.* 2010; Klingenberg 2015). Typical examples of such structures are symmetric body parts (Klingenberg and McIntyre 1998; Klingenberg *et al.* 2002). The degree of fluctuating asymmetry (FA), i.e. random asymmetric deviations in otherwise symmetric structures, is often used as a measure of DI, and it has been proposed as an indicator of both environmental and genetic stress (Graham *et al.* 2010).

Studies of FA in symmetric flower parts have shown that there may be multiple sources of variation, both intrinsic and environmental, that lead to elevated FA levels. Various fitness-related environmental features, such as pollution (Møller and Shykoff 1999), elevated atmospheric CO_2_ (Andalo *et al.* 2000) or increased illumination (Tucić and Miljković 2010), were found to be correlated with different estimates of flower FA based on linear measurements. Lower fitness of individuals with more asymmetric flowers was also explicitly illustrated in *Epilobium angustifolium*, where higher rates of embryo abortion were related to increased FA (Møller 1996). However, it should be noticed that published evidence for the relationship between the FA of flower parts and fitness-related characteristics of plants is not unambiguous, and several studies did not find any such relationship in various plant species (Waldmann 2001; Siikamäki *et al.* 2002; Barišić Klisarić *et al.* 2016). Likewise, Barišić Klisarić *et al.* (2019) reported only a weak relationship between the pollution from highway traffic and shape FA in flowers of *Iris pumila*.

Plants are sessile organisms, which means that individual symmetric parts of flowers may be differently affected by directed environmental heterogeneity, such as solar irradiation. This effect was recently demonstrated in flowers of *I. pumila*, which showed shape FA related to compass direction indicating varying levels of light availability for different parts of flowers (Tucić *et al.* 2018). It should be noted that this part of shape FA cannot be attributed to DI but rather to phenotypic plasticity expressed by the organism in response to directed environmental effects. In addition to environmental factors, shape FA in flowers can also be significantly elevated by intrinsic processes, such as inbreeding (Waldmann 2001; Sandner 2020) or hybridization between populations (Waldmann 1999).

However, we are not aware of any previous study looking for the sexual differentiation of flowers as a possible source of FA in the shape of symmetric flower parts. Our hypothesis was that corollas of female flowers might have increased DI due to lower selective pressure for their morphological symmetry and possible developmental perturbations caused by stamen abortion. Recently, it was shown that the position and shape of petals composing the corolla of female flowers in gynodioecious *Euonymus europaeus* were more asymmetric than their hermaphroditic counterparts (Neustupa 2020). However, in their purely actinomorphic flowers, typical of radial symmetry lacking any adaxial–abaxial differentiation, it was not possible to differentiate between possible directional asymmetry (DA) and FA of their corolla shapes. Nevertheless, these results still indicated that purely pistillate flowers of *E. europaeus* are typical for a less orderly arrangement and shape of their petals in comparison with hermaphroditic flowers. Thus, similar scenarios in the zygomorphic corolla with bilateral symmetry might be indicated by different levels of shape FA between the sexes.

For this study, we chose the zygomorphic corolla shapes in *Glechoma hederacea*, a species typical for relatively stable gynodioecy and well-documented size dimorphism between female and hermaphroditic flowers (Plack 1957; Widén 1992; Hutchings and Price 1999). The hermaphroditic flowers of this species are typical for high pollen fertility that solely ensures the male function within populations (Widén and Widén 1990; Widén 1992). The pistillate flowers lack any pollen production and their fertilization entirely depends on pollen transfer from the hermaphrodites (Widén 1992; Godin 2014). The principal questions of the study were focused on the symmetric shape variation and FA between the sexually differentiated flowers. First, we asked if there is any difference in the amount of shape FA between the left and right halves of the corolla lower lip in pistillate and hermaphroditic flowers. In addition, the secondary question asked for the differences in the amounts of shape variation among flowers in each of the two sexually differentiated groups. Thus, the null hypotheses are that average shape differences among flowers and bilateral shape FA within each sexual group are approximately identical. These questions were evaluated using geometric morphometric analyses of corolla shapes of *G. hederacea*, including the analysis of their bilateral symmetry and asymmetric components.

## Materials and Methods

### Study species

**Figure 1.**
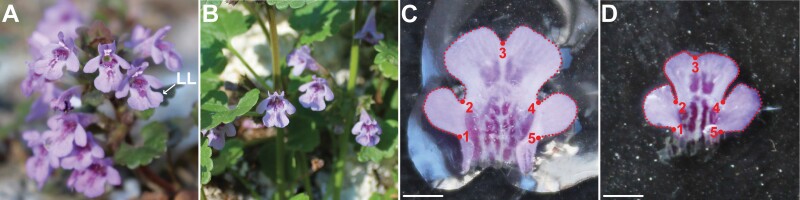
Flowers of *Glechoma hederacea* and the lower lips of corolla with five LMs and four curves spanned by 29 equidistant semi-landmarks placed along the outlines. (A) The hermaphroditic ramet with flowers including fertile stamens; (B) the female ramet with purely pistillate flowers; (C) the lower lip of a hermaphroditic individual; (D) the lower lip of a female individual. LL: lower lip. Scale (C, D) = 2 mm.

*Glechoma hederacea* is a widely distributed perennial herb native to Europe and Asia (Hutchings and Price 1999; Chrtek 2000). In addition, it has been considered an aggressive invasive species of various habitats in North America and New Zealand (Hutchings and Price 1999). In Central Europe, it is one of the most frequently occurring members of the family *Lamiaceae*, inhabiting both semi-natural and anthropogenic habitats, such as gardens, urban parks, mesotrophic grasslands and forest margins (Sádlo *et al.* 2007). In the lowland regions of Central Europe, flowers of this species appear in early April, and the flowering period usually lasts for 6–8 weeks. The flowers are characterized by a zygomorphic corolla composed of two bilaterally symmetric lips (Fig. 1). The lower lip, composed of three joined petals, is considerably larger than the comparatively inconspicuous upper lip. Visually, the lower lip is the single most striking structure of the flowers, divided into a large central lobe (CL) and two lateral lobes (LLs), providing the landing platform for insect pollinators (Hutchings and Price 1999; Godin 2014). Similar to numerous other members of Lamiaceae, *G. hederacea* is a gynodioecious species with most individuals bearing either hermaphroditic or purely pistillate flowers (Plack 1957; Widén and Widén 1990; Widén 1992). Occasional gynomonoecious individuals, bearing flowers of both sexes, have also been reported (Widén 1992). Hermaphroditic flowers are the sole providers of pollen produced by four didynamous stamens. In female flowers, the stamens are either rudimentary and almost completely obliterated or reduced into sterile staminodes (Hutchings and Price 1999; Widén and Widén 2000).

### Sampling

The flowers of *G. hederacea* were sampled at two localities in Central Bohemia, Czech Republic. While the first locality consisted of ~1 ha of an old garden and orchard area at 316 m a.s.l. (50.195816N, 14.031799E), the second sampling site was in the Knovíz village at 238 m a.s.l. and consisted of the village park and parkway (50.212517N, 14.136929E). Sampling was performed on 20–23 April 2020, when the populations were at the peak flowering phase (Fig. 1A and B). Individual ramets were sampled at least 20 m from one another. In total, 120 randomly selected flowers from 12 plants (six females, six hermaphrodites) were sampled. The sexual identity of each ramet was determined by inspecting multiple flowers along the stem in different flowering phases. The fertility of stamens in hermaphrodite flowers was checked by inspecting the presence of pollen in the locules. Likewise, the sterility of staminodes in female flowers was determined by inspecting the lack of any pollen in their rudimentary anthers. A stereomicroscope Zeiss SM1 (Carl Zeiss, Jena, Germany) was used for these observations.

Flowers were picked at anthesis, and the intact corolla was immediately pulled out of the calyx. Then, the upper lip was carefully cut off with a scalpel. The lower lip was then placed on a black surface without any additional compression or flattening (Fig. 1C and D). This allowed for the reproducible acquisition of two-dimensional corolla representations without any artificial deformation of fragile forms of these zygomorphic flowers. The photographs were made at a fixed distance of 20 cm using a Canon EOS 1200D (Canon Inc., Oita, Japan) digital camera with an EFS 18–55 mm objective. To assess imaging error, each object was photographed twice independently by different persons in different rotations.

### Data acquisition

The studied forms were digitized by the combination of five fixed landmarks (LMs) and four curves placed along the outlines (Fig. 1C and D). Landmarks 1 and 5 were placed at the points of the crossover of the LL outline with the corolla tube. Landmarks 2 and 4 depicted the position of the incision between the CL and LLs. Finally, LM 3 was placed at the apical incision of the CL. Each of the four curves consisted of 29 equidistant points placed along the outline limited by two consecutive, fixed LMs. Thus, these curves depicted the outlines of the two LLs and both CL halves.

The fixed LMs were digitized manually in TpsDig, ver. 2.22 (Rohlf 2015) and the outline points were generated by the semi-automatic *draw-background-curve* function of this software. Digitization was performed by the first author. Equidistant position of the points relative to fixed LMs was assured by the function *digit.curves* of the package *geomorph*, ver. 3.3.1 (Adams and Otárola-Castillo 2013), in R., ver. 4.0.2 (R Core Team 2020). To assess digitization error, all objects were registered twice. In the first run, LMs and curves were registered clockwise, starting from the left margin of the objects. The second digitization proceeded counterclockwise from the opposite starting point. Then, the points from the later digitization were re-labelled to match the labels of the clockwise digitization.

### Geometric morphometrics of bilateral symmetry

Geometric morphometric analysis of bilaterally symmetric structures required two symmetry transformations of the original coordinates prior to generalized Procrustes analysis (GPA): (i) identity and (ii) reflection of the coordinates across the axis of symmetry (Klingenberg *et al.* 2002; Savriama 2018). This reflection also required re-labelling of individual points that were opposite to each other across the axis of bilateral symmetry. Thus, in addition to the fixed LMs, each of the four curves swapped their locations with those placed in the mirror positions across the symmetry axis. Following these steps, the resulting data set consisted of 120 (original flowers) × 2 (photographs of each flowers) × 2 (digitizations of each photograph) × 2 (symmetry transformations) = 960 configurations.

The equidistant points delimiting the curves were treated as semi-landmarks (Bookstein 1997; Gunz and Mitteroecker 2013). This means that GPA involved additional steps of iterative sliding of these points along the outline tangents to achieve their final position, yielding the smoothest possible deformation of each configuration to the mean shape of the data set. Such positions of these points were defined by the lowest possible bending energy between each configuration and the mean shape (Perez *et al.* 2006). GPA with sliding semi-landmarks was carried out using the function *gpagen* implemented in the package *geomorph*, ver. 3.0.7.

Procrustes-aligned coordinates were used as the dependent variable in the multivariate type I analysis of variance (ANOVA) model evaluating shape variation between two sexual groups, among the plants and flowers, the asymmetric components of DA and FA and the components of measurement error (Klingenberg 2015). The analysis decomposed the matrix of Procrustes distances among individual configurations into different sources specified by the independent factors. The significance of these effects was tested by randomization tests based on the comparison of Procrustes sum of squares (SS), spanned by individual factors with the distribution of random SS yielded by 999 permutations (Schaefer *et al.* 2006). The permutation strategy reflected the nested structure of the data. Thus, the main fixed effect of ‘sex’ was evaluated by random reshuffling of plants between two sexual groups. Likewise, the SS spanned by the ‘plant’ effect nested within ‘sex’ was evaluated against the random distribution yielded by reshuffling of flowers among plants within the sexual groups. Then, the effect of individual flowers was tested against the random SS distribution based on the reshuffling of the original and mirrored configurations among different flowers. The effect of ‘reflection’ quantifying the bilateral DA, i.e. the average asymmetry between the left and right halves of the studied structure, was tested against the random distribution of Procrustes SS yielded by coin-flipping of the side assignment (original or reflected/re-labelled) of the configurations within individual flowers. Finally, the interaction effect ‘reflection:flower’ accounted for the individual asymmetric deviations denoting the shape FA of configurations. The SS of this effect was evaluated by comparison with the random SS obtained by reshuffling individual configurations among the flowers and their side assignments. The function *procD.lm*, in package *geomorph*, ver. 3.0.7, was used for this analysis. The shapes typical for each sexual group were visualized in TpsRegr, ver. 1.42 (Rohlf 2015), as the displacement of individual points from the consensus configuration.

Symmetric configurations were obtained by averaging the Procrustes-aligned original and mirrored versions of individual flowers. Symmetric shape variations within each sexual group were quantified by Procrustes distances of each configuration to the respective consensus shape. The configurations representing shape FA were acquired from the residuals of the multivariate linear model after accounting for the effects of symmetric variation among flowers and DA represented by the ‘reflection’ effect. Residual values were added to the original consensual configuration of the data set and averaged across the repetitions due to double imaging and digitization. Then, individual FA was estimated as the Procrustes distance from the consensual configuration (Klingenberg 2015).

Differences in the intersexual levels of symmetric shape variation among flowers and their FA were evaluated by bootstrap and permutation analyses of the mean values of both sexual groups. The overlap of the 95 % confidence intervals of these values between the sexual groups was taken as the indication that the null hypothesis of equal means should not be rejected (Good 2005). In parallel, significance of the differences in the mean values was compared by the permutation *t*-test with 999 random repetitions. These randomization tests were conducted in PAST, version 2.17c (Hammer *et al.* 2001).

### Principal component analysis and multivariate regression

Principal component analysis (PCA) was used to visualize the principal trends in the patterns of shape change, both in the symmetric and asymmetric subspaces. Thus, PCA of the symmetrized configurations yielded principal components (PCs) that illustrated the shape changes equally affecting both halves of the studied structure. Conversely, shape changes spanned by the asymmetric axes affected the mirrored parts of the configurations in the opposite way.

Centroid size, the square root of the sum of squared distances of all LMs and semi-landmarks from their centroid (Klingenberg 2015), was used as a size measure of the configurations. Then, the relationship of symmetric shape variation and centroid size of the objects was evaluated by a separate multivariate ANOVA model based on the matrix of Procrustes distances among the specimens as the dependent variable and centroid size as the sole independent factor. The analysis was conducted using the function *procD.lm*, implemented in the package *geomorph*, ver. 3.0.7. Shapes typical for the extremes of the resulting allometric regression line were visualized in TpsRegr, ver. 1.42. The differences in centroid size between both sexual groups were evaluated by the bootstrap and permutation analyses analogous to those conducted with the values of shape symmetry and FA.

The main patterns of asymmetric variation were illustrated by PCA of the residuals of the multivariate ANOVA model, representing shape FA (Klingenberg *et al.* 2002). Consequently, the resulting PCs showed the purely asymmetric variation involving opposite shape changes in corresponding LMs located in the bilaterally symmetric halves of the structure. Shapes typical for marginal occupied positions at individual PCs of both morphospaces were visualized in TpsRelw, ver. 1.65 (Rohlf 2015).

## Results

### Symmetric shape variation and size differences

**Figure 2.**
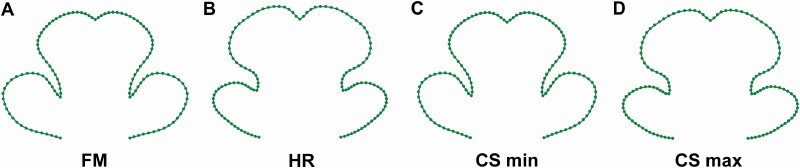
Lower lip shapes reconstructed by multivariate regression models. (A, B) Characteristic shapes of the female (FM) and hermaphroditic (HR) flowers; (C, D) characteristic shapes of the smallest (CS min) and largest flowers (CS max) in the data set. The differences between the sexual morphs are two times enhanced for better visibility.

**Figure 3.**
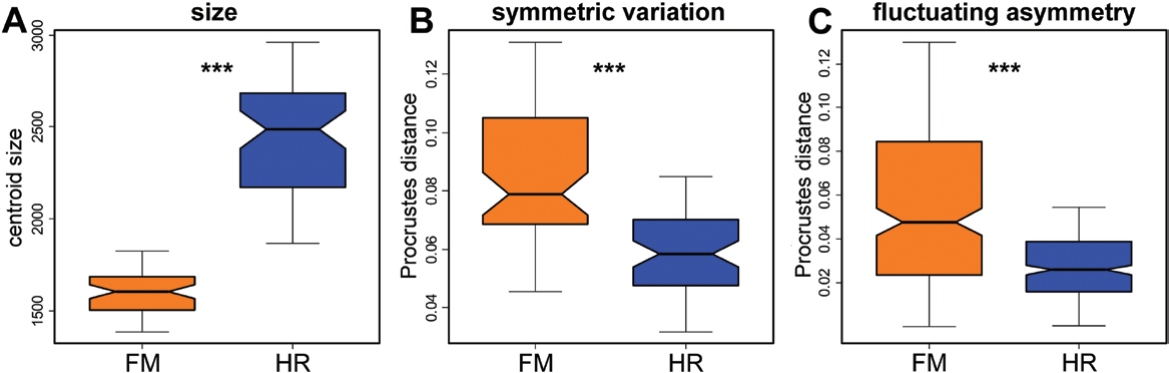
Comparison of (A) size, (B) symmetric shape variation and (C) FA in the female and bisexual flowers of *G. hederacea. P*-values of the two-group permutation *t*-tests for the difference in means are shown: ***, 0.001. FM (orange): female flowers; HR (blue): hermaphroditic flowers.

The corolla lower lip shapes of the purely pistillate flowers were considerably different from those of the hermaphrodites. In particular, the female flowers were typical for relatively larger LLs and more compressed CL in comparison to the flowers of the hermaphroditic individuals (Fig. 2A and B). This difference was reflected by the significant effect of ‘sex’ in the multivariate linear model that tested for shape variation spanned by this factor against the differences among individual plants (Table 1). In total, the effect of sexual differentiation accounted for 10.1 % of the symmetric and asymmetric shape differences in the studied data set. These intersexual shape differences were also accompanied by pronounced variation in size. The corollas of the female flowers were ~34.6 % smaller than those of the hermaphroditic flowers and this difference was highly significant at the in the permutation *t*-test with *P*-value = 0.001 (Fig. 3A; Table 2). In parallel, the multivariate linear model fitting the overall symmetric shape variation to centroid size of the objects described 12.9 % of the total variation (df = 1, SS = 0.119, MS = 0.119, *F* = 17.62, *P*-value = 0.001). The shapes typical for both margins of the allometric regression line were very similar to those reconstructed for sexual morphs (Fig. 2C and D). Thus, the smallest flowers in the data set were typical for features corresponding to the female flowers and the largest flowers had the shape features of the hermaphrodites.

**Table 1.** Results of multivariate analysis of variance evaluating variation in shape of the corolla lower lip in female and hermaphroditic flowers of *Glechoma hederacea*. df: degrees of freedom; SS: sum of squares; MS: mean squares; η ^2^: coefficient of determination; *P*: probability of the null hypothesis.

Source	df	SS	MS	η ^2^	*P*
Sex	1	0.9673	0.96728	0.1014	0.019
Plant (sex)	10	3.4821	0.34821	0.3649	0.001
Flower (plant)	108	3.2435	0.03003	0.3399	0.001
Reflection	1	0.0219	0.02191	0.0023	0.151
Reflection: flower	119	1.6513	0.01388	0.1730	0.001
Digitizing error	240	0.0864	0.00036	0.0091	
Imaging error	240	0.0511	0.00021	0.0054	
Digitizing: imaging	240	0.0401	0.00017	0.0042	
Total	959	9.5437			

**Table 2.** Results of bootstrap tests for the difference in means of size and shape parameters between the female (FM) and bisexual (HR) flowers. *P*-value refers to probability of equal means based on 999 permutations of the data. CI: confidence interval; SEM: standard error of the mean.

Factor	Mean (FM)	SEM (FM)	95 % CI for mean (FM)	Mean (HR)	SEM (HR)	95% CI for mean (HR)	*P*-value
Centroid size	1583.4	19.203	[1547.8, 1621.0]	2420.2	39.658	[2343.1, 2497.9]	0.001
Symmetric variation	0.0910	0.0047	[0.0816, 0.0996]	0.0601	0.0024	[0.0553, 0.0648]	0.001
Fluctuating asymmetry	0.0471	0.0033	[0.0406, 0.0533]	0.0276	0.0011	[0.0253, 0.0298]	0.001

**Figure 4.**
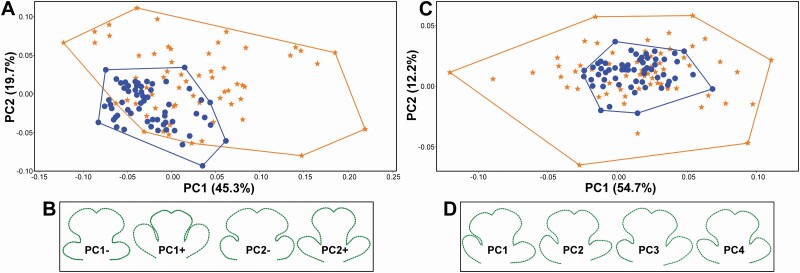
Principal component analysis (PCA) ordination plot of (A) symmetric variation and (B) shape trends associated with first two PCs. The configurations illustrate shapes typical for the opposite most marginal occupied positions within the shape space. (C) PCA ordination plot of bilateral FA in shape of the lower lips and (D) shape trends typical for PC1–4. In asymmetric axes each configuration depicts one of the mirror shapes located at the opposite positions within the shape space. FM (orange asterisks): female flowers; HR (blue circles): hermaphroditic flowers.

Differences in average symmetric shapes of the corolla lower lips among individual plants within the sexes were also strongly significant (*P*-value = 0.001; Table 1). Notably, this single effect spanned almost 36.5 % of the total shape variation in the studied data set. Likewise, symmetric differences in the shape of the 120 analysed flowers proved to be highly significant against the random distribution (Table 1). Interestingly, the female flowers contributed more to this effect as they were considerably more variable among one another than the hermaphroditic flowers (Fig. 3B; Table 2). This pattern was also apparent from the position of individual flowers on the first two PCs yielded by PCA of the symmetric variation (Fig. 4A). These two most important PCs described the shape trends involving coordinated changes in all three parts of the structure. Thus, PC1 explained 45.3 % of the symmetric shape variation and it illustrated differences between the shapes typical for a prominent CL and small LLs separated by broadly opened incisions and those with extremely large LLs laterally appressed to a relatively smaller CL (Fig. 4C). In parallel, PC2 described 19.7 % of the symmetric variation and it highlighted the differences between shapes with broadly expanded CL and short LLs from those typical by narrower CL and somewhat elongated LLs (Fig. 4C).

### Directional and fluctuating asymmetry

Shape DA (i.e. average asymmetry between the left and right halves of the configurations) was very subtle and it only accounted for 0.23 % of the total shape variation. The observed SS for this effect proved to be well within the random distribution range of SS values created by coin-flipping of the side assignments of the configurations (Table 1). Thus, the null hypothesis of the lack of any shape DA of the lower lips could not be rejected.

The interaction term ‘reflection:flower’ accounted for individual asymmetric deviations between the left and right halves representing shape FA. Although this asymmetric term explained considerably less variation than the symmetric differences among flowers and it only accounted for 17.3 % of the total variation, it was highly significant at *P*-value = 0.001 in comparison to the components of measurement error (Table 1). The purely pistillate flowers proved to be considerably more asymmetric than the hermaphrodites (Fig. 3C; Table 2). This trend was again clearly visible from the ordination plot, illustrating the most prominent patterns of shape FA (Fig. 4B). These asymmetric PCs were typical for the mirror position of the original and reflected/re-labelled configurations representing a single flower. Their distance from the symmetric consensus configuration located at the centroid of the ordination space reflects the amount of shape asymmetry in different patterns described by individual PCs. We can see that the hermaphroditic flowers were clearly less asymmetric regarding the variation spanned by the first two PCs, typical of trends largely involving varying asymmetry in arrangement and shape of the left and right LLs (Fig. 4D).

## Discussion

Our analyses confirmed the clear-cut differences in size of the corolla structures between female and hermaphrodite flowers of *G. hederacea*. The observed differences in centroid size of the corolla lower lips were in line with earlier studies evaluating different linear size dimension measures of sexually differentiated flowers of *G. hederacea* (Plack 1957; Widén and Widén 1990; Godin 2014) or other gynodioecious members of Lamiaceae (Balant *et al.* 2019; Zhang and Claßen-Bockhoff 2019). Several studies reported that besides stable hermaphroditic and female individuals, populations of *G. hederacea* also included a small fraction of transitional ramets with partially male-sterile flowers and intermediate corolla size (Widén 1992; Widén and Widén 2000). However, we did not sample any such individuals, and consequently, the analysed flowers were unambiguously divided into two sexual groups with clear differences in corolla size.

In parallel to the differences in size, the sexually differentiated flowers also varied in shape. These differences were apparent both as significant shape distance between the mean symmetric shapes of two sexes, as well as the unequal amounts of symmetric and asymmetric shape variation within the groups. It should be noted that the statistical power of the permutation test evaluating significance of the symmetric shape differences between the sexes was limited due to the low number of cases within the nested subgroup in the hierarchical structure of the analysed data. However, the single binary factor of ‘sex’ accounted for >10 % of the total variation and the corresponding shape differences were closely similar to the allometric shape dynamics reconstructed by the multivariate regression analysis (Fig. 2). Thus, it is likely that the observed intersexual shape differences were part of the allometric shape-to-size relationship across the size range of the studied corollas.

The purely pistillate flowers were considerably more plastic, based on their higher shape distances among each other, and asymmetric, as illustrated by their higher FA levels. In other words, morphogenesis of the male-sterile flowers was typical for decreased developmental stability, enhancing both symmetric variation and bilateral FA of the female corollas at the population level. The female and hermaphrodite individuals of *G. hederacea* inhabit the same habitats in their natural localities (Widén 1992; Godin 2014). Therefore, there are no reasons to expect any differences in environmental heterogeneity of their immediate surroundings causing higher variation of the female flowers. Thus, these differences are rather linked to weaker stabilizing selection for the shape of the purely pistillate corollas, facilitating their relatively higher symmetric shape variation and asymmetry. The prominent lower lip of corollas in *G. hederacea* forms a dominant visual attractor for pollinators approaching the flowers head-on and from above. Several studies have illustrated the innate preference of different insect pollinators, such as honeybees and bumblebees, for bilateral flower symmetry (Moller and Sorci 1998; Rodríguez *et al.* 2004; Wignall *et al.* 2006), which also correlates with higher pollen placement during individual insect visits (Culbert and Forrest 2016). In addition, in *Erysimum mediohispanicum*, a species with a continuous range of corolla morphology ranging from radial to bilateral symmetry, the flowers with zygomorphic shapes were comparatively more attractive for pollinators than actinomorphic or strongly asymmetric flowers (Gómez *et al.* 2006). Thus, in a gynodioecious system with bilaterally symmetric corollas, such as *G. hederacea*, the natural selection imposed by preferences of pollinators for bilateral symmetry may lead to higher developmental stability of hermaphroditic flowers ensuring the male function within populations. In parallel, lower selective pressure for pollination frequency in females may then allow for their more variable flowers to be kept in the populations.

Our analyses illustrated that most of the asymmetric variation was concentrated to the differences in shape and position of two LLs. Interestingly, the lateral sides of the lower lips in the genus *Lamium* are used as the supporting platform for legs of the visiting insects (Sulborska *et al.* 2014). Therefore, it is possible that conspicuous differences in shape asymmetry of LLs between female and hermaphroditic flowers of *G. hederacea* also indicate their role in pollination visits. This would mean that these two lobes are subjected to relatively stronger selection in the pollen-bearing flowers leading to their comparatively higher developmental precision. It has been known since the 1950s that corolla development in *G. hederacea* is closely linked with the presence of functional stamens in flowers (Plack 1957; Delph 1996). Experimental removal of anthers from the buds of hermaphroditic individuals resulted in the development of female-sized corollas (Plack 1957). In general, this close developmental relationship between the stamens and corolla has recently been explained by the activity of B-class genes of the ABCE model that are simultaneously involved in the development of these flower parts (Chanderbali *et al.* 2016; Theißen and Rümpler 2018). Thus, it is likely that increased shape variation and DI of the zygomorphic female corollas, illustrated in this study, could be linked to this developmental pattern. Likewise, increased deviations from the radial symmetry of tetramerous actinomorphic corollas in the male-sterile flowers of gynodioecious *E. europaeus* also pointed to a similar developmental causation (Neustupa 2020). Thus, it is possible that the decrease in corolla symmetry of the male-sterile flowers is a general pattern among angiosperms. This raises several testable hypotheses that could be evaluated in various angiosperm models. If the lack of functional male organs generally leads to increased corolla shape asymmetry, then it should also be detectable in the purely pistillate flowers of monoecious and dioecious taxa with full separation of the sexes among flowers. Conversely, the evolutionary digression from dioecy leading to androdioecious or andromonoecious taxa with either hermaphroditic or female-sterile flowers (Barrett 2002) should lack any such differences in FA of corolla shape. We believe that these hypotheses open up an interesting field for future research in microevolutionary patterns of corolla shape variation, which might be tested using geometric morphometric analyses of suitable model taxa within angiosperms.

Geometric morphometrics of biological symmetry has become established as a powerful methodology for evaluation of various evolutionary, ecological or developmental hypotheses (Klingenberg 2015; Graham and Özener 2016). However, vast majority of the published studies used animal or human systems and the use of modern methods of shape analysis in the research of flower symmetry has only recently become more frequent in floral biology (Gómez and Perfectti 2010; Frey and Bukoski 2014; Tucić *et al.* 2018; Sandner 2020). This study has shown for the first time that geometric morphometrics of bilateral symmetry was useful in detection and quantification of previously unknown subtle differences in shape and symmetry of the sexually differentiated zygomorphic flowers in a well-known and widely distributed plant species. However, the outcomes are restricted by limited sample size and narrow geographical scope of the study. For example, it is possible that varying composition of pollinators or different environmental conditions across distribution area of the studied species might significantly modify the observed patterns. Thus, more general conclusions on the evolutionary and ecological dynamics of shape symmetry in sexual differentiation of flowers should only be based on the extensive data sets with broader geographic and taxonomic scope.

In summary, our study has shown that, in addition to dimorphism in size, the gynodioecious corollas of the studied populations of *G. hederacea* are also characterized by shape dimorphism, leading to increased variation and left–right asymmetry of female flowers. Thus, besides fitness-related characteristics (Møller and Shykoff 1999; Andalo *et al.* 2000; Tucić and Miljković 2010), compass orientation (Tucić *et al.* 2018) or inbreeding (Waldmann 2001; Sandner 2020), sexual differentiation of flowers needs to be considered as an additional factor affecting shape FA of flower parts in natural populations of angiosperms.

## Data Availability

Primary data and the R scripts used for the analyses are available online at: http://doi.org/10.5281/zenodo.4567439.

## Notes

**Form & Function. Chief Editor:** Kate McCulloh
